# Hierarchical Cluster-based Partial Least Squares Regression (HC-PLSR) is an efficient tool for metamodelling of nonlinear dynamic models

**DOI:** 10.1186/1752-0509-5-90

**Published:** 2011-06-01

**Authors:** Kristin Tøndel, Ulf G Indahl, Arne B Gjuvsland, Jon Olav Vik, Peter Hunter, Stig W Omholt, Harald Martens

**Affiliations:** 1 Centre for Integrative Genetics (CIGENE), Dept. of Mathematical Sciences and Technology, Norwegian University of Life Sciences, P. O. Box 5003, N-1432 Ås, Norway; 2 Auckland Bioengineering Institute, The University of Auckland, 70 Symonds Street, Auckland, New Zealand; 3 CIGENE, Dept. of Animal and Aquacultural Sciences, Norwegian University of Life Sciences, P. O. Box 5003, N-1432 Ås, Norway

## Abstract

**Background:**

Deterministic dynamic models of complex biological systems contain a large number of parameters and state variables, related through nonlinear differential equations with various types of feedback. A metamodel of such a dynamic model is a statistical approximation model that maps variation in parameters and initial conditions (inputs) to variation in features of the trajectories of the state variables (outputs) throughout the entire biologically relevant input space. A sufficiently accurate mapping can be exploited both instrumentally and epistemically. Multivariate regression methodology is a commonly used approach for emulating dynamic models. However, when the input-output relations are highly nonlinear or non-monotone, a standard linear regression approach is prone to give suboptimal results. We therefore hypothesised that a more accurate mapping can be obtained by locally linear or locally polynomial regression. We present here a new method for local regression modelling, Hierarchical Cluster-based PLS regression (HC-PLSR), where fuzzy *C*-means clustering is used to separate the data set into parts according to the structure of the response surface. We compare the metamodelling performance of HC-PLSR with polynomial partial least squares regression (PLSR) and ordinary least squares (OLS) regression on various systems: six different gene regulatory network models with various types of feedback, a deterministic mathematical model of the mammalian circadian clock and a model of the mouse ventricular myocyte function.

**Results:**

Our results indicate that multivariate regression is well suited for emulating dynamic models in systems biology. The hierarchical approach turned out to be superior to both polynomial PLSR and OLS regression in all three test cases. The advantage, in terms of explained variance and prediction accuracy, was largest in systems with highly nonlinear functional relationships and in systems with positive feedback loops.

**Conclusions:**

HC-PLSR is a promising approach for metamodelling in systems biology, especially for highly nonlinear or non-monotone parameter to phenotype maps. The algorithm can be flexibly adjusted to suit the complexity of the dynamic model behaviour, inviting automation in the metamodelling of complex systems.

## Background

Realistic deterministic dynamic models of complex biological systems are difficult to assess with respect to behaviour, since they are characterised by a large number of parameters and state variables as well as nonlinear functional relationships and intricate feedback loops. The relationship between the output from such models and their input parameter variation and initial conditions, and control of built-in functional relationships, can become extremely complex. The traditional analytical tools of nonlinear systems theory are mainly aimed at studying the effects of few parameters on low-dimensional phenomena such as steady states and limit cycles. The high-dimensional models emerging in systems biology bring new challenges such as increasing the speed of numerical solvers, developing tools for automated model simplification and global sensitivity analysis (study of how the system input variations influence the output [1]). Metamodels of a dynamic model, which are statistical models mapping parametric variation to variation in the state variables throughout the entire relevant parameter space [2], will be helpful in meeting several of these challenges.

There is currently little tradition for making use of metamodelling in advanced computational biology, but based on experiences from other fields [2,3] we anticipate that this is up for change. Since computationally demanding multiscale modelling is an emerging field in computational biology, we foresee that metamodel-generated mappings will be very useful as a model reduction technique for speeding up simulations, for performing global high-dimensional sensitivity analyses for a whole range of purposes, for comparing the high-dimensional prediction spaces of competing models as well as for comparing models to high-dimensional phenotypic data. Input parameters and initial conditions can also be predicted from the model output or empirical data, providing opportunities for identification of biologically relevant parameters. However, for these anticipations to be fulfilled we need to develop a robust metamodelling methodology capable of producing accurate predictive mappings for a whole range of different biological models and which allows for extensive automation. Despite that considerable progress has been made in other fields, in particular engineering [1-6], there is a need for substantial development to make this approach generally applicable in biology and feasible for implementation e.g. as part of the model repositories like the CellML repository [7-9].

Most metamodelling efforts published to date make use of ordinary least squares (OLS) regression and response surface methods based on OLS that require the covariance matrix of the regressors (independent variables) to be invertible [2], that is, the regressors must be linearly independent. These methods are primarily focused on prediction of one *single *output variable at a time, and usually ignore strong inter-correlations between the output variables. An exception is Artificial Neural Networks (ANN), but these methods produce models of a format that do not allow easy interpretation. Using a Bayesian approach, Conti and O'Hagan [10] recently demonstrated that it is possible to emulate the output of a dynamic model to a high degree of precision using only a few hundreds of training runs. However, such Gaussian emulators are only effective for model structures having a small number of significant main effects and very mild interactions [1]. Hence, one may claim that none of the current metamodelling approaches do make transparent how *all *inputs, auxiliaries and outputs are related to each other *jointly *under *a broad range of different conditions*. They are also, with few exceptions, unable to utilise inter-correlations between the output variables. Furthermore, according to Li *et al*. [11], most sensitivity analysis methods require the regressor variables to be linearly independent, something that is not likely to be the case in many biological modelling situations (e.g. due to the use of highly reduced experimental designs or sampling-based methods to set up the computational experiments). Hence, there is a need for new methodologies for mapping the entire space of biologically relevant parameters and initial conditions to the outcome of the corresponding dynamic models, that can handle linearly dependent input parameters, utilise strong inter-correlations between the outputs, as well as model highly nonlinear and non-monotone input-output relations, which characterise many biological systems. Mild nonlinearities can to some degree be modelled by polynomial regression, using e.g. square- and interaction terms as extra regressors [12], but a robust metamodelling methodology must be capable also of handling strong nonlinearities, in particular non-monotone input-output relationships [13-15].

A candidate approach is to make use of locally linear or locally polynomial regression modelling of carefully selected subsets of the input-output space of the original complex model. Locally linear approaches to modelling large and complex data sets have been successfully demonstrated in a variety of applications, most of which include a separation of the data into blocks based on prior knowledge about the structure of the data being analysed [16-20]. This suggests that nonlinear and non-monotone response surfaces can be handled by local high-order polynomial models. In order to also account for linearly dependent regressors and inter-correlations between the responses, alternatives to OLS are needed. Bi-linear methods based on estimated latent variables, e.g. Principal Component Analysis (PCA) [21,22] and Partial Least Squares Regression (PLSR) [23-25] (see Additional file 1: Appendix 1), have shown considerable success in analysis and interpretation of a large number of highly multivariate and complex data sets. Martens and Martens [26] demonstrated the use of PLSR as an alternative to Analysis Of Variance (ANOVA) to facilitate the interpretation of multi-response data from designed experiments. PLSR maximises the explained covariance between the regressors and the responses. In contrast to most other linear regression methods, PLSR also utilises inter-correlations between the response variables for model stabilisation, and does not require the regressor variables to be linearly independent. PLSR is efficient for compressing inputs, intermediate states and output variables into their most relevant subspaces (spanned by the estimated latent variables, also called PLS components (PCs)), and hence provides a versatile means for data compression by reducing the rank of both regressors (X) and responses (Y). This, in turn, provides an effective approach to identification of important features in a complex system. PLSR is equivalent to OLS when the regressor rank is not reduced, that is, when all PLS components are included. Modelling based on such estimated latent variables (represented by so-called X- and Y- score vectors) also have the advantage of being suited for graphical visualisation, inspection and interpretation via their associated sets of loadings, i.e. the coefficients describing the relationship between the score vectors and the original variables/parameters. Theoretical aspects of PLSR and its relations to the more commonly used method Principal Component Regression (PCR) [27,28] are given in [29]. Campbell *et al*. [30] have shown that metamodels based on subspaces found by PLSR, when compared to Legendre polynomials and PCA, gave the simplest and most predictive basis for sensitivity analysis for a set of computational models. In [31], the suitability of PLSR for interpretation of complex biological systems and use of PLSR in sensitivity analysis was demonstrated. This motivated us to probe the versatility of a new variant of local modelling, here named Hierarchical Cluster-based PLS regression (HC-PLSR), which assumes no prior knowledge about the data structure. Besides presenting the HC-PLSR method, we report the metamodelling performance of HC-PLSR, global PLSR and global ordinary least squares regression on three dynamic model systems in order of increasing complexity: i) six different gene regulatory network models with various types of feedback [32,33], ii) a dynamic model of the mammalian circadian clock [34] and iii) a model of the mouse ventricular myocyte function [35]. These three test beds encompass large classes of dynamic models. We show that the HC-PLSR approach is superior in all cases and that the difference in terms of explained variance and prediction accuracy increases with the degree of nonlinearity and the presence of positive feedback loops.

## Methods

### *In silico *data sets

#### Gene regulatory networks

**Figure 1:**
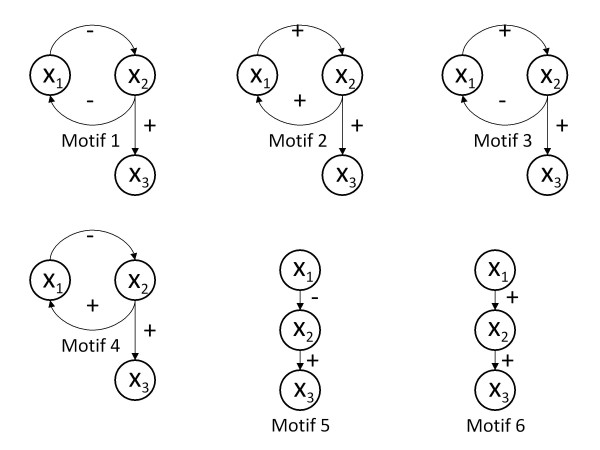
**Connectivity diagrams for the six simulated gene regulatory network motifs**. The dynamics of the three state variables (X1, X2 and X3) were computed in six different gene regulatory network motifs with different feedback systems [32,33].

Gene regulatory networks were modelled using the sigmoid formalism [36,37]. In this formalism the state variable Xi denotes the expression level of gene i, and the i-th differential equation describes production and decay of the gene product as well as regulation of these processes by other genes in the network (see [32] for a more detailed description). The dynamics of three state variables (X1, X2 and X3) were computed over 300 time steps in six different gene regulatory network motifs with different feedback systems [32,33]. The initial conditions were varied in a full factorial design (FFD) with five equally spaced levels for each, resulting in 125 different starting conditions between 0 and 2 for each state variable. Since differences in feedback loops lead to qualitatively different behaviour (e.g. positive feedback can give rise to multistationarity) they should also lead to large differences in the nonlinear terms in the regression analysis. We therefore used five levels of each factor in order to explore these nonlinear terms thoroughly. The parameter values of maximal production rates, decay rates and regulation thresholds were constant, and chosen to ensure that the steady state levels for all three variables were ranging from 0 (when the production rate is zero) to 2 for a maximal production rate. For each of the gene regulatory network motifs, the time series data for the three state variables were concatenated into a matrix representing 125 starting conditions × 900 time points, corresponding to the set of 125 × 3 initial conditions. The connectivity diagrams for the six simulated gene regulatory network motifs are shown in Figure 1, and illustrate that Motif 1 and 2 have positive feedback, Motif 3 and 4 have negative feedback and Motif 5 and 6 have no feedback. The simulations were carried out in MATLAB^® ^Version 7.9.0.529 (R2009b) [38], using in-house code that can be obtained from the authors upon request.

#### Mammalian circadian clock

The simulations of the mammalian circadian clock were generated using a model developed by Leloup and Goldbeter [34]. The ordinary differential equation (ODE) model describes a mammalian consensus system of three key genes: Bmal1, Per and Cry which regulate circadian rhythms by means of intertwined positive and negative feedback loops. The model consists of 16 coupled differential equations with state variables representing mRNA, nonphosphorylated and phosphorylated proteins for the three genes, as well as protein complexes. The original publication [34] contains four different parameter sets and we used parameter set 4 (corresponding to continuous darkness) as the basal parameter set. A curated CellML implementation [7-9] of the model was downloaded from http://models.cellml.org. The parameter combinations used to generate the calibration set were generated using an Optimised Multi-level Binary Replacement (OMBR) Design [39,40] of 9 factors (see Table 1) with 8 equally spaced levels each. This resulted in 8192 simulations with the circadian clock model. A full factorial design of 9 factors at 8 levels each would result in 8^9 ^> 134 million runs. Hence, the OMBR design was chosen, in order to explore the effects of as many factors as possible while revealing possible nonlinear effects of parameters on phenotypes. This was considered important in this example, since the mammalian circadian clock model is widely used and explored previously, and an optimisation study is therefore most relevant. Detailed insight into the nonlinear behaviour of the model is obtained using a large number of levels of each factor. In the OMBR design method, the values of the original parameters are replaced by multi-bit binary representations, and the binary factor bits are then combined in a fractional factorial design according to the chosen confounding pattern. Thereby drastically reduced experimental designs are obtained, yet maintaining reasonable coverage of the parameter space. The OMBR design used here was optimised by establishing a number of alternative binary confounding patterns, and choosing the one that best satisfied the quadratic D-optimality criterion. This OMBR design has been compared to central composite designs and random sampling, and has been shown to have good predictive ability [40]. The range of each parameter is given in Table 1.

**Table 1:** Description and range of the parameters varied in the simulations with the mammalian circadian clock model

Parameter name	Unit	Description	Minimum value	Level step size	Maximum value
v_mB_	nMh^-1^	Maximum rate of Bmal1 mRNA degradation	0.16	0.017	0.28

v_mC_	nMh^-1^	Maximum rate of Cry mRNA degradation	0.80	0.086	1.40

v_mP_	nMh^-1^	Maximum rate of Per mRNA degradation	0.88	0.094	1.54

v_dPCN_	nMh^-1^	Maximum rate of degradation of nuclear phosphorylated Per-Cry complex	0.80	0.086	1.40

v_dIN_	nMh^-1^	Maximum rate of degradation of nuclear Per-Cry-Clock-Bmal1 complex	0.64	0.069	1.12

k_1_	h^-1^	Rate constant for entry of the Per-Cry complex into the nucleus	0.64	0.069	1.12

k_3_	nM^-1^h^-1^	Rate constant for the formation of the Per-Cry complex	0.64	0.069	1.12

k_5_	h^-1^	Rate constant for entry of the Bmal1 protein into the nucleus	0.32	0.034	0.56

k_7_	nM^-1^h^-1^	Rate constant for the formation of the inactive Per-Cry-Clock-Bmal1 complex	0.40	0.043	0.70

For each parameter combination the resulting differential equation model was solved from the original initial conditions (see [34]) until convergence to a stable limit cycle. The differential equations were solved in SUNDIALS 2.3 [41] using a wrapper for PySundials http://pysundials.sourceforge.net. The resulting data set consists of values of 16 state variables (corresponding to the 16 differential equations in the model) calculated over 200 time steps each for the set of 8192 combinations of values of the nine input parameters. The test for convergence was done as follows: First the system was solved with rootfinding for variable M_B _to extract two complete cycles. Convergence of the cycle period was tested by requiring that the period difference relative to the mean of the periods for the two cycles should be less than 0.001. Convergence to synchronous oscillations was tested by (i) interpolating all 16 state variables at 200 equally spaced time points for each cycle, (ii) linearly transforming each state variable such that the minimum and maximum values of each cycle was 0 and 1, respectively, and (iii) requiring that the sum of absolute difference between the two cycles across all the 3200 interpolated time points should be less than 0.0001.

#### Mouse ventricular myocyte

Data for the murine heart cell function was generated using the model recently published by Li *et al*. [35], built to account for the action potential and calcium transient of the cardiomyocyte in terms of its constituent ion currents and gating channels. The model extends that of Bondarenko *et al*. [42], with more realistic calcium handling, detailed re-parameterisation to experimental data and consistency checking by conservation of charge. State variables include concentrations of sodium, potassium and calcium in the cytosol, calcium concentration in the sarcoplasmic reticulum and the state distribution of ion channels. Formulated as a system of 36 coupled ODEs, this model provides a comprehensive representation of membrane-bound channels and transporter functions as well as fluxes between the cytosol and intracellular organelles.

The integration was carried out in CVODE [43], which is part of the SUNDIALS 2.3 package [41], using in-house code that can be obtained from the authors upon request. Ten different parameters (see Table 2) were varied in an FFD with three levels of each parameter (baseline value ± 50%), resulting in 59049 simulations. Because the parameter space of the mouse heart cell model has been less extensively explored than that of the mammalian circadian clock, we designed for screening rather than optimisation here. Screening designs are typically used to identify which factors are most important, while optimisation studies aim for a more detailed insight into the relationships between the various factors and the response. The range of each parameter is given in Table 2. The data set resulting from the mouse ventricular myocyte function simulations consists of values of 36 state variables and 81 auxiliary variables (notably including ion currents that can be monitored and manipulated in patch clamp experiments (see [35])) calculated over 100 time steps (one period) each for the set of 59049 combinations of values of the ten input parameters. The time steps were adaptively chosen by the solver (Vik JO, Gjuvsland AB, Li L, Tøndel K, Smith N, Hunter PJ, Omholt SW: Genotype-phenotype map characteristics of an in silico heart cell, Submitted). The pacing protocol was a stimulus setting the transmembrane potential to -15 mV for 3 ms. This was repeated until convergence or a maximum of 1000 stimuli. The convergence criterion for each state variable was based on its value at the beginning of each interval and the integral of its trajectory over that interval, both being constant to within a relative tolerance of 0.001. A running history of ten intervals was kept, and after each interval we checked for a match against the previous ones. Cell dynamics was categorised as "failed" if dynamics did not converge to period 1 within 1000 stimulus intervals. Details of alternans were not pursued.

**Table 2:** Description and range of the parameters varied in the simulations with the mouse ventricular myocyte model

Parameter name	Unit	Description	Minimum value	Baseline value	Maximum value
Ko	uM	Extracellular potassium concentration	2700	5400	8100

Nao	uM	Extracellular sodium concentration	67000	134000	201000

Cao	uM	Extracellular calcium concentration	700	1400	2100

stim_period	ms	Stimulus period	166.67	333.33	500

vmup_init	uM/ms	SERCA, calcium uptake from cytosol to sarcoplasmic reticulum	0.2530	0.5059	0.7589

P_CaL	1/ms	L-type calcium current	1.25	2.5	3.75

V_max_NCX	pA/pF	NCX, scaling coefficient for the sodium-calcium exchanger current	1.9695	3.9390	5.9085

g_Na	mS/uF	Fast sodium current	8	16	24

g_K1	mS/uF	Time-independent potassium current	0.1750	0.35	0.5250

g_Kr	mS/uF	Rapid delayed rectifier potassium current	0.0083	0.0165	0.0248

Only the state variables were included in the regression analysis (not the auxiliary variables), and the constant state variable iKss was omitted. For this data set 15793 of the 59049 simulations (26.7%)) failed, and were therefore omitted in the analysis. In order for the time series to be comparable, interpolation to a fixed set of time points was needed. For the entire time series to be analysed, the data set had to be separated into three parts, according to the stimulus period (the values of the stimulus period are given in Table 2). The time series for each part of the data set were then interpolated using the MATLAB^® ^[38] system function "interp1.m" into 200 equally spaced time points between 0 and the stimulus period.

### Metamodelling procedures

Implementation of the HC-PLSR was based on an initial global second order polynomial PLSR using all observations in the calibration set with a 10-fold cross-validation (i.e. the data was separated into ten randomly chosen segments, and ten PLSR models were made where one segment was successively taken out and predicted using a PLSR model based on the other nine segments) to identify a preliminary (global) model (mean of the ten models made in the cross-validation). The global model (i.e. the optimal number of PLS components (PCs)) was then chosen according to the minimum cross-validated mean squared error (MSE) of prediction of the response matrix Y, with the extra requirement that each included component accounts for at least 1% of the total cross-validated Y-variance. Using cross-validation to find the optimal rank is a well established technique to minimise the risk of overfitting. When modelling empirical data, the portion of the variance accounted for by the omitted PCs are assumed to be caused by noise. However, in this case, we used data from deterministic modelling which is free from noise, but since we aimed for an as simple and easily interpretable metamodel as possible (but showing adequate predictive ability), we regarded variations not accounted for by a second order polynomial as stochastic noise and used cross-validation to truncate the model. The cross-terms and second order terms were calculated from mean-centred parameter values.

Thereafter the observations were clustered by fuzzy *C*-means (FCM) clustering [44,45] of the X- or Y-scores using Euclidian distance for a chosen number of PLS components (X is the regressor matrix, while Y is the response variable matrix). This allowed separation of the overall data set into subsets where local polynomial regression was hypothetically more likely to improve prediction results. In our MATLAB^® ^implementation the FCM fuzzifier parameter was chosen equal to 2 (a standard choice). The fuzzifier parameter can be interpreted as an increased sharing of points among all clusters. Fuzzy clustering was chosen because the FCM algorithm is simple, efficient and less prone to local minima than crisp clustering algorithms. The FCM algorithm is also flexible with respect to the distance measure used, and it is easy to incorporate various types of penalties in the distance measure [45]. It is therefore a good choice in making the HC-PLSR method widely applicable.

**Figure 2:**
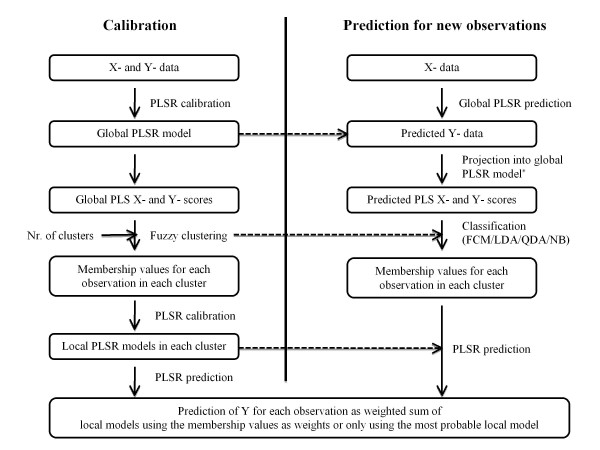
**Illustration of the HC-PLSR approach**. The HC-PLSR "pipeline" starts with calibration of an initial global polynomial PLSR using all observations in the calibration set. This global PLSR model provides PLS scores and loadings, which constitute the basis for separation of the calibration set observations into groups by fuzzy *C*-means (FCM) clustering [44,45]. Local PLSR models are then calibrated in each cluster. Predictions of response variables for new observations (or test set observations) are done by a) selecting the local model for the most probable cluster based on classification or by b) computing the regression coefficients as a weighted sum of the local models, where the weights are estimated cluster membership probabilities from the classification. *See Additional file 1: Appendix 1, Eq. A4 and A6.

To prevent possibly unstable regression models due to a small number of calibration observations, we post-processed the clustering with the requirement that each cluster should contain at least ten observations. Smaller clusters (and their associated observations) were regarded as outliers, and not included in the subsequent local regression modelling. Local second order polynomial PLSR models were finally calibrated individually for each of the remaining clusters, using 10-fold cross-validation to find the optimal number of PLS components in the same manner as in the global modelling. Using polynomial PLSR instead of OLS (corresponding to PLSR using the maximal number of PLS components) is an advantage in hierarchical regression modelling, especially for large data sets, since with OLS one would have to cluster on the regressor matrix (X) itself, instead of the response (Y)-relevant X-scores. This would be much more computationally demanding than clustering on the reduced rank X-scores. In addition to this, OLS requires the regressor covariance matrix to be invertible, that is, the regressor variables need to be linearly independent. Even in cases where the original regressor matrix is generated using an experimental design method that ensures that the design variables are linearly independent in the full data set, this may still not be true within the clusters used in the local regression modelling. The MATLAB^® ^[38] function "plsregress.m" (from the Statistics Toolbox™ v7.2) was used for the PLSR both for the global and the local regression analyses. Figure 2 gives an overview of the HC-PLSR "pipeline". The MATLAB^® ^code can be obtained from the authors upon request.

New observations (or test set observations) were classified based on X- or Y-scores calculated by projection into the global PLSR model. X-scores for test set observations were calculated based on loading weights from the global PLSR model (see Additional file 1: Appendix 1, Eq. A4). If Y-scores were used in the clustering, the global PLSR model was used for prediction of Y to acquire the Y-scores from the loading weights of the global PLSR model (see Additional file 1: Appendix 1, Eq. A4 and A6). Four different ways of classifying an observation into the appropriate cluster were compared: i) the nearest cluster centre from the FCM clustering (based on Euclidian distance), ii) Linear Discriminant Analysis (LDA) [46], iii) Quadratic Discriminant Analysis (QDA) [47] and iv) Naive Bayes (NB) classification [47]. We used the MATLAB^® ^functions "classify.m" and "NaiveBayes.m" from the Statistics Toolbox™ v7.2 for classification options ii)-iv), and in-house MATLAB^® ^code for option i).

Predictions of response variables for the test set observations were done both by a) selecting the local model for the most probable cluster and by b) computing the regression coefficients as a probability-weighted sum of the local regression models. Outlier-check of new observations was based on the Euclidian distance matrix from the clustering of all non-outliers in the calibration set (based on the X- or Y-scores depending on what was used for clustering and classification). The maximal occurring distance from the cluster centre was found for each cluster by inspection of the calibration results, and 1.5 times these values were used as outlier limits for the respective clusters. Prediction of the outliers (corresponding to no appropriate local model) was by convention handled by the global PLSR model.

To further improve the prediction accuracy we also considered an alternative HC-PLSR approach based on hierarchical regression modelling of the residuals from the global PLSR model. Local modelling was here used only on the Y-residuals (still using the original regressor matrix), and the final predicted Y was the sum of the global predictions and the local predictions. The clusters were based on the global X-scores as described above, and we therefore used the same number of clusters as for the hierarchical regression modelling of the entire Y-matrix. This gave a small gain in prediction accuracy (the results for the mammalian circadian clock data are shown in Additional file 1: Appendix 3, Figure A3.4). However, this approach was not chosen since some interpretability is lost due to the more complex Y-loadings based on the Y-residuals.

### Evaluation of the metamodelling performance of HC-PLSR

The three test applications were chosen in order to represent a wide range of dynamic model types, in order to obtain a valuable evaluation of the metamodelling performance of HC-PLSR in systems biology. This is important in order for the method to be generally applicable e.g. as part of model repositories like the CellML repository [7-9]. The first model setting represents very simple models, where differences between various types of feedback can be explored. The two other model settings represent more complex models with many parameters and state variables, connected through various types of feedback loops. As described above, model setting 2 and 3 represent an optimisation study and a screening study, respectively.

In the applications described above, a set of parameter combinations and state variable initial conditions were used as input to an ODE-based dynamic model, and the output was a set of state variables calculated over a number of time steps. In metamodelling of the dynamic models, the parameter values and initial conditions were then used as regressors (X) and the state variable time series were used as response variables (Y) in a regression analysis, in order to predict the state variable time series directly from the parameters and initial conditions.

Second order polynomial HC-PLSR (including interaction terms and second order terms in both the global and local regression models) was compared to global, second order polynomial PLSR (using 10-fold cross-validation to optimise the number of PLS components) and global second order polynomial OLS (equivalent to PLSR were all PLS components are used) with respect to test set prediction accuracy. Polynomial PLSR is equivalent to Implicit Non-linear Latent Variable Regression (INLR) [12], except that in INLR cross-terms are not included. Table 3 gives an overview of the variables included in the regressor- and response matrices and shows which test sets were used in the three application examples. The same data were used with all three regression methods. The squared correlation coefficient (R^2^) and Root Mean Squared Error of Prediction (RMSEP) values for the state variable time series of the test set observations were used as measures of prediction accuracy.

**Table 3:** Overview of the regressor- and response matrices used in the regression analyses and the test sets used for the three application examples

Application	Design variables (D)	Regressor matrix (X)	Response matrix (Y)	Test set used
Gene regulatory networks	125 initial conditions for the three state variables X1, X2 and X3 (at time zero) in a 5^3 ^FFD (dimensions: 125 × 3)	[D sin(D) cos(D)]	The concatenated time series for the state variables X1, X2 and X3 (Y_i _= 3 × 300 time points, i = 1 to nr. of observations)	33% of the observations in D (randomly chosen), and the corresponding Y-values

Mammalian circadian clock model	Nine model parameters in an OMBR design using eight levels for each parameter (dimensions: 8192 × 9)	[D cross-terms of D D^2^]	16 state variable time series modelled separately (for each state variable Y_i _= 200 time points, i = 1 to nr. of observations)	8192 new parameter combinations generated by random Monte Carlo sampling (here the entire matrix D was used for calibration) and corresponding Y-values

Mouse ventricular myocyte model	Ten model parameters in a 3^10 ^FFD (dimensions: 59049 × 10)	[D cross-terms of D D^2^]*	35 state variable time series modelled separately (for each state variable Y_i _= 200 time points, i = 1 to nr. of observations)*	33% of the observations in D that did not fail (randomly chosen), and the corresponding Y-values

*The three parts of the mouse ventricular myocyte data set separated according to the stimulus period were analysed separately, and only the simulations that did not fail were used.

In HC-PLSR, only the local model corresponding to the most probable cluster for each observation was used in the test set prediction, since this approach gave slightly better results than using a weighted sum of all local models. The appropriate numbers of HC-PLSR clusters were chosen by inspection of the R^2 ^and RMSEP values of Y within the calibration set, resulting from predictions using a range of different numbers of clusters. Here the calibration set observations were first used for calibration and subsequently treated as "new observations" and classified prior to prediction, that is, the same procedure as for the test set was used in the prediction stage. The minimum number of clusters in HC-PLSR giving (approximately) maximum obtained predictive ability (maximal R^2 ^and minimal RMSEP) was chosen for each state variable time series.

#### Model setting 1: Gene regulatory networks

In metamodelling of the six gene regulatory networks, the initial values of the state variables were used as regressors (X) (125 starting conditions × 3 state variables) and for each gene regulatory network, the concatenated time series for the state variables X1, X2 and X3 were used as response variables (Y) (3 state variables × 300 time steps = 900 Y-variables). The state variable data generated for the gene regulatory network motifs are shown in Additional file 1: Appendix 2, Figure A2.1. Sine and cosine terms (in radians) of X were included in the regressor matrix, while cross-terms and second order terms were omitted here. This was the result of a "trial and error" procedure to choose between different types of nonlinear terms to include in the regression equation. This could be afforded here due to the relatively small size of the data set. Adding sine and cosine terms to the regressor matrix turned out to be advantageous in order to model the nonlinearities present in this data set. All regressor and response variables were mean-centred prior to the regression analysis.

In the HC-PLSR, FCM clustering on the Y-scores was chosen due to the low number of X-variables (initial conditions for three state variables), and the fact that these were derived by a full factorial design. FCM clustering on the first three PCs of the Y-scores was used to establish the local PLSR models, since this gave a very distinct clustering of the observations. QDA based on globally predicted Y-scores for new observations gave the best classification results for this data set, and was therefore used for the test set prediction, using 33% of the observations (randomly selected from the FFD) as a separate test set (the rest of the observations were used for calibration). The test set was chosen randomly from all possible combinations of parameter values in order to be representative for the population.

#### Model setting 2: Mammalian circadian clock

In metamodelling of the mammalian circadian clock model, the state variable time series (Y) were modelled as functions of the parameters of the dynamic model and their cross- and second order terms (X). Each of the time series for the 16 state variables (see Additional file 1: Appendix 3, Figure A3.1) was modelled separately, giving 16 regression models for each regression method. Each regression model could thus predict the response in 200 time steps for one of the 16 state variables. The state variable time series (response variables, Y) were log-transformed, and all variables were mean-centred prior to the regression analysis.

FCM clustering on the X-scores from the global PLSR model was used in the HC-PLSR, and the classification of new observations was based on the distances to the cluster centres (in predicted X-scores for new observations) from the fuzzy clustering. Clustering on the X-scores is in general more safe compared to using the Y-scores, since the Y-scores for new observations to be predicted have to be calculated from predicted Y-values from the global PLSR model. The X-scores are calculated from the X-data for new observations, and their accuracy is therefore not dependent on the quality of the global PLSR model. However, only the first three PLS components of the X-scores were used for clustering, in order for the clusters to be as relevant as possible for both Y and X (the first PCs explain the largest amount of the covariance between X and Y).

The circadian clock test set consisted of 8192 new parameter combinations generated by random Monte Carlo sampling [48,49] from a uniform probability distribution within the same parameter ranges as for the calibration set. Hence, the test set had the same size as the calibration set. The reason why we chose to generate a separate test set based on Monte Carlo sampling instead of sampling randomly from the original design as in the gene regulatory network example is that here a highly reduced design was used instead of an FFD. A test set sampled from a highly reduced design is less likely to be fully representative for the population than a test set sampled from an FFD (which includes all possible combinations of parameter values). Furthermore, sampling from the OMBR design to generate a test set would reduce the calibration set further, and the calibrated regression model would be less representative. Since the OMBR is optimised with respect to quadratic D-optimality [40], using part of the design as test set would also make the calibration set less optimal for inclusion of second order polynomial terms in the regression analysis.

#### Model setting 3: Mouse ventricular myocyte

In metamodelling of the mouse ventricular myocyte model, the state variable time series (Y) were modelled as functions of the parameters of the dynamic model and their cross- and second order terms (X), using the same procedure as for the mammalian circadian clock model. Each of the time series for the 35 state variables (see Additional file 1: Appendix 4, Figure A4.1) was modelled separately. The parameters were mean-centred and standardised (divided by their standard deviations) due to the large differences in absolute values for the parameters, and the state variables were mean-centred prior to the regression analysis.

In this study, 33% of the observations (randomly selected from the simulations in the FFD that did not fail) were used as a separate test set. The rest of the observations were used for calibration. The data set was separated according to the stimulus period, and the regression calibration and test set predictions were carried out for each part of the data set separately. The average R^2 ^and RMSEP values over the three data set parts were then calculated for each state variable.

## Results

### Model setting 1: Gene regulatory networks

**Figure 3:**
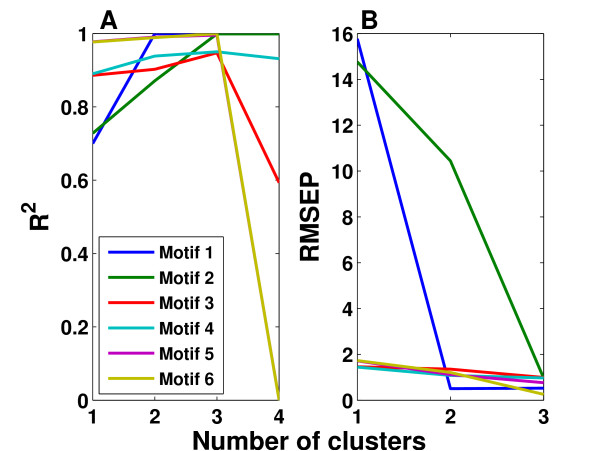
**Selection of the optimal number of clusters in HC-PLSR for the gene regulatory network motifs**. A) R^2 ^and B) RMSEP values from calibration set predictions of the gene regulatory network time series using from 1 to 10 clusters in HC-PLSR. The calibration set observations were first used for calibration of the HC-PLSR model and subsequently treated as "new observations" and classified prior to the predictions. Each line represents one gene regulatory network motif. The X-axes are truncated since RMSEP increases enormously when the number of clusters is larger than 3. One cluster is equivalent to ordinary PLSR.

**Figure 4:**
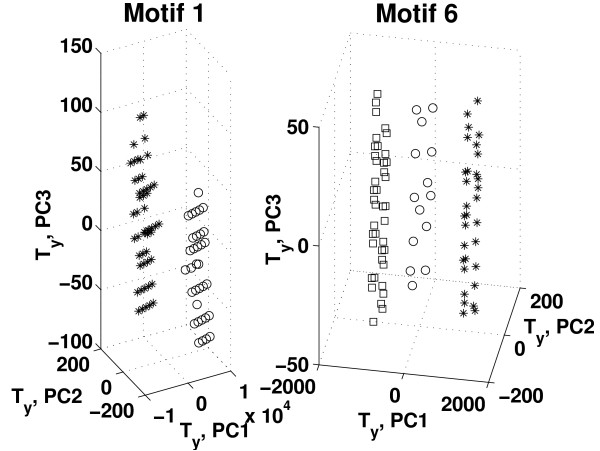
**Clustering results used in HC-PLSR for gene regulatory network motif 1 and 6**. The global first three PLSR Y-scores (PC1-PC3) obtained for the calibration set data from gene regulatory network motif 1 and 6. Stars: cluster 1, circles: cluster 2, squares: cluster 3 (for motif 6). The range within which the starting conditions for the three state variables X1, X2 and X3 varied in each cluster is given in Additional file 1: Appendix 2, Table A2.1.

Based on the results in Figure 3, HC-PLSR with two clusters was chosen for Motif 1, while for Motif 2-6 using three clusters was considered to be optimal. Figure 4 illustrates the clustering results obtained from the global PLSR Y-scores prior to the HC-PLSR calibration for gene regulatory motif 1 and motif 6. The range within which the starting conditions for the three state variables X1, X2 and X3 varied in each cluster used in the HC-PLSR modelling of gene regulatory network motif 1 and 6 are shown in Additional file 1: Appendix 2, Table A2.1.

**Figure 5:**
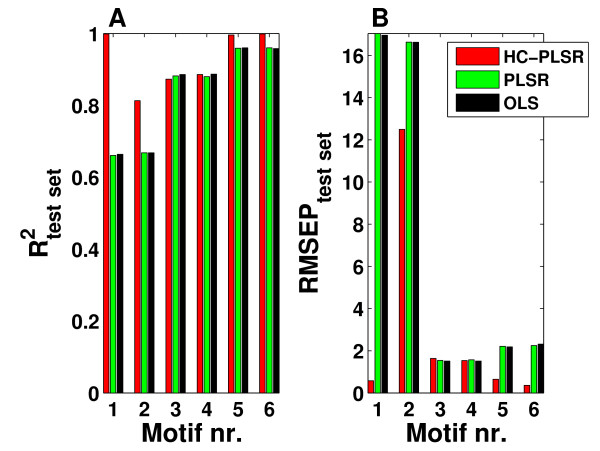
**Test set prediction results from HC-PLSR, PLSR and OLS for the gene regulatory networks**. A) R^2 ^and B) RMSEP values from test set predictions of the gene regulatory network state variable time series as functions of the starting conditions, with HC-PLSR, global PLSR and global OLS as grouped bars. In HC-PLSR, 4-9 PLS components were used in the regression models, while in PLSR 3-6 PLS components were used. The mean R^2^_test set _over all motifs was 0.93 for HC-PLSR and 0.84 for both PLSR and OLS.

The prediction results from HC-PLSR, PLSR and OLS on the separate test set for the gene regulatory network data are shown in Figure 5, and show that the HC-PLSR approach performs better than both global PLSR and OLS in modelling the state variable time series from their initial conditions, especially for the gene regulatory network motifs containing positive feedback loops (Motif 1 and 2). The mean R^2^_test set _over all motifs was 0.93 for HC-PLSR and 0.84 for both PLSR and OLS.

When clustering on the Y-scores as is done for the gene regulatory motif data set, a relatively good global PLSR model is needed for reasonable prediction of the Y-scores for the test set. In this example, the lowest global model R^2^_test set _value (0.66) was obtained for gene regulatory network motif 1. This is good enough for a reasonable Y-scores prediction for this data set, since HC-PLSR results in an R^2^_test set _value of 0.99 (see Figure 5). It should, however, be kept in mind that the global model needs to provide some reasonable information to be used for prediction of the Y-scores. The required quality of the global model depends on how distinct the separation of the clusters is. Less distinctly separated clusters need a higher quality of the Y-scores predictions for the Y-scores to give reliable classification results.

### Model setting 2: Mammalian circadian clock

**Figure 6:**
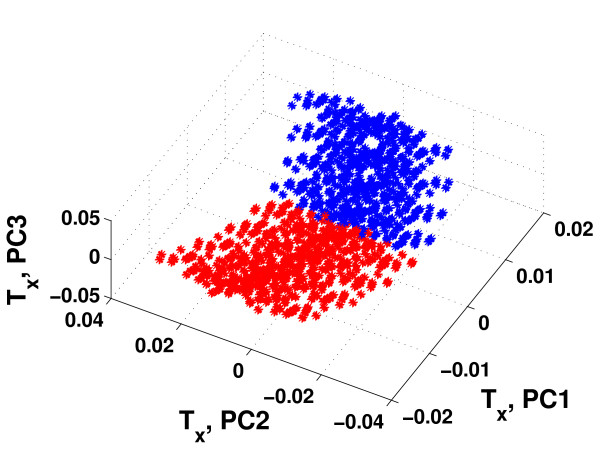
**Clustering results used in HC-PLSR for the mammalian circadian clock state variable M_p_**. The global first three PLSR X-scores (PC1-PC3) obtained for the calibration set data for the mammalian circadian clock state variable M_P_. The observations are coloured according to the two-cluster solution used in the HC-PLSR (blue: cluster 1, red: cluster 2).

**Figure 7:**
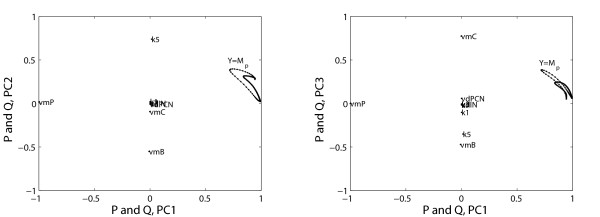
**Correlation loadings for the mammalian circadian clock state variable M_p_**. The global PLSR X- and Y-correlation loadings (P and Q, respectively) for PC1 to PC3 obtained for the calibration set data for the circadian clock state variable M_P_. X-loadings (model parameters) are shown as dots, while Y-loadings (time series) are shown as a curve. The loadings for the cross-terms and second order terms of the parameters are not shown in order to increase the overview.

The optimal number of clusters to be used in HC-PLSR for the circadian clock data set was found to be 15 or 20 for most of the state variables (see Additional file 1: Appendix 3, Figure A3.2), but for two of the state variables a two-cluster solution was sufficient. Figure 6 shows the global PLSR X-scores and corresponding clustering result (coloured) for the observations prior to the HC-PLSR calibration for the state variable M_P_, while Figure 7 shows the corresponding global X- and Y- correlation loadings (see Additional file 1: Appendix 1). Figure 7 shows that loading plots are useful for illustration of the relations between X- and Y-variables in the estimated latent variable space, and indicates that the parameter v_mP _accounts for the variation in PC1, and is negatively correlated with the state variable M_P _time series (Y). Hence, globally, v_mP _has a large, negative effect on the state variable M_P_, since PC1 accounts for the largest part of the covariance between X and Y. The parameters v_mB _and k_5 _account for most of the variation in PC2, while PC3 is made up of v_mC_, v_mB _and k_5_.

**Figure 8:**
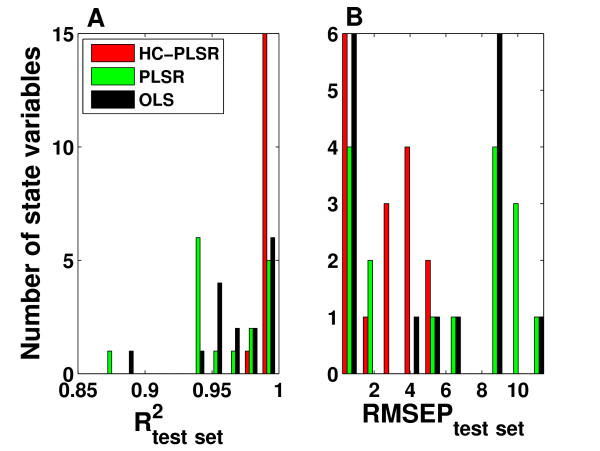
**Test set prediction results from HC-PLSR, PLSR and OLS for the mammalian circadian clock**. Histograms of A) R^2 ^values and B) RMSEP values from test set predictions of the mammalian circadian clock state variable time series as functions of the parameters, with HC-PLSR, global polynomial PLSR and global polynomial OLS. In HC-PLSR, 4-19 PLS components were used in the regression models, while in PLSR 3-17 PLS components were used. The mean R^2^_test set _over all state variables was 0.99 for HC-PLSR, 0.96 for PLSR and 0.97 for OLS.

The accuracy of the test set predictions for the three regression methods is compared in Figure 8 as histograms of R^2 ^and RMSEP values for the 16 state variables, indicating that HC-PLSR is superior to global polynomial PLSR and global polynomial OLS with regard to predictions of the state variable time series. The mean R^2^_test set _over all state variables was 0.99 for HC-PLSR, 0.96 for PLSR and 0.97 for OLS. The HC-PLSR results in R^2^_test set _values larger than 0.98 for all state variables (Figure 8). The prediction results from OLS and PLSR are less robust. For the state variables P_C_, P_CP_, PC_C_, C_C_, C_CP_, PC_CP_, PC_N_, PC_NP _and I_N _(see Additional file 1: Appendix 3, Figure A3.1), HC-PLSR works much better than PLSR and OLS. In light of that HC-PLSR excels at positive feedback motifs for the gene regulatory networks in model setting 1, and that these state variables are all involved in a large positive feedback loop (see [34]), a possible explanation is that the presence of the positive feedback loop is the reason why HC-PLSR gives much more accurate results than PLSR and OLS for these state variables. As shown in Additional file 1: Appendix 3, Figure A3.3, most observations in the test set have relatively low prediction residuals from HC-PLSR, and the mean prediction error (taken over the 200 time steps) decreases as the distance from the nearest cluster centre decreases (increasing membership value for most probable cluster).

### Model setting 3: Mouse ventricular myocyte

**Figure 9:**
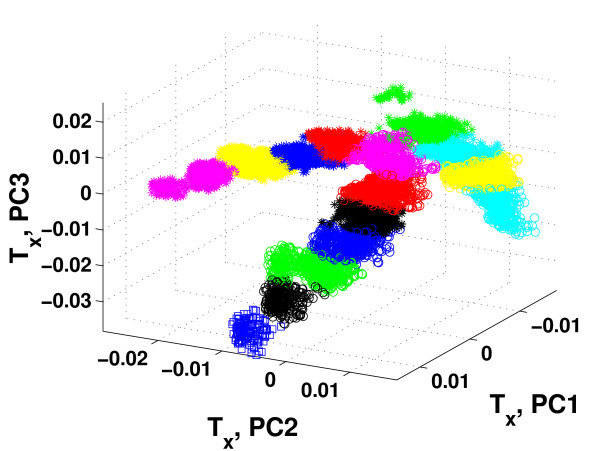
**Clustering results used in HC-PLSR for the mouse ventricular myocyte state variable V**. The global first three PLSR X-scores (PC1-PC3) obtained for the calibration set data for the mouse ventricular myocyte state variable V, the action potential (333.33 ms stimulus period data set). The observations are coloured according to the 15 clusters to be used in the HC-PLSR.

**Figure 10:**
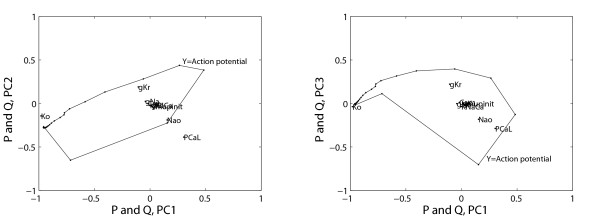
**Correlation loadings for the mouse ventricular myocyte state variable V**. The global PLSR X- and Y-correlation loadings (P and Q, respectively) for PC1 to PC3 obtained for the calibration set data for the mouse ventricular myocyte state variable V (the action potential). X-loadings (model parameters) are shown as dots, while Y-loadings (time series) are shown as a curve. The loadings for the cross-terms and second order terms of the parameters are not shown in order to increase the overview.

The optimal number of clusters to be used in HC-PLSR was found to be between 10 and 20 for most state variables in the mouse ventricular myocyte data set (see Additional file 1: Appendix 4, Figure A4.2). Figure 9 shows the global PLSR X-scores and corresponding clustering result (coloured) for the observations used in the HC-PLSR calibration for the state variable V (the action potential), while Figure 10 shows the corresponding global X- and Y- correlation loadings. For simplicity we show only the results for the part of the data set having a stimulus period of 333.33 ms (baseline value). Figure 10 indicates that the parameters having the largest impact on the mouse ventricular myocyte action potential (V) are Ko, P_CaL, Nao and g_Kr.

**Figure 11:**
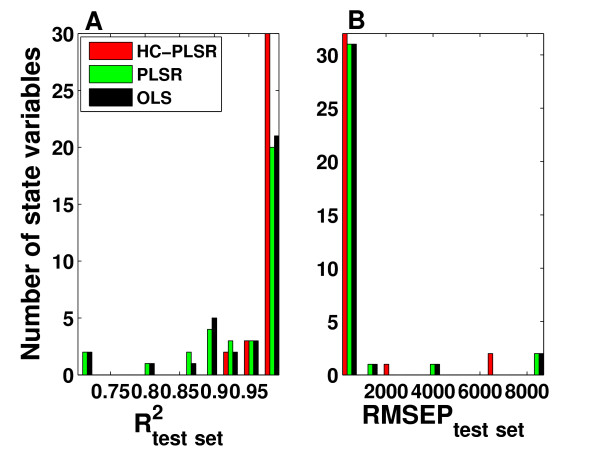
**Test set prediction results from HC-PLSR, PLSR and OLS for the mouse ventricular myocyte**. Histograms of A) R^2 ^values and B) RMSEP values from test set predictions of the mouse ventricular myocyte state variable time series as functions of the parameters, with HC-PLSR, global polynomial PLSR and global polynomial OLS. The average values over the three data set parts (separated according to the stimulus period) are shown. In HC-PLSR, 2-20 PLS components were used in the regression models, while in PLSR 2-8 PLS components were used. The mean R^2^_test set _over all state variables was 0.98 for HC-PLSR and 0.94 for both PLSR and OLS.

The test set prediction results from HC-PLSR, PLSR and OLS on the murine heart cell data (average over the three data set parts separated according to the stimulus period) are shown in Figure 11. Also for this data set, HC-PLSR results in a higher prediction accuracy than the other two regression methods in prediction of the state variable time series. The mean R^2^_test set _over all state variables was 0.98 for HC-PLSR and 0.94 for both PLSR and OLS. Figure 11 shows that the results from HC-PLSR are good for all state variables. As shown in Additional file 1: Appendix 4, Figure A4.3, most observations have relatively low HC-PLSR prediction residuals. The results from global polynomial PLSR and OLS are less robust. This indicates that there are nonlinearities or non-monotone input-output relations that a global polynomial regression model is not able to model correctly, but that local modelling by HC-PLSR can account for. The difference between the results from HC-PLSR and the other two regression methods is largest for the state variables P_O1_, P_C2_, O_Na_, C_Na1 _and C_Na2_, perhaps due to a higher degree of nonlinearity and/or a non-monotone response surface (see Additional file 1: Appendix 4, Figure A4.1).

## Discussion

HC-PLSR provides a very flexible metamodelling methodology, since the number of local models (clusters) can be adjusted and the model adapted to suite the complexity of the response surface of the dynamic model. Hence, even very complex data sets containing a large number of different types of input-output relations can be quite accurately modelled through modelling each type of response surface separately. HC-PLSR using only one cluster is equivalent to a global PLSR model. In principle, the least complex among several types of regression methods leading to good predictions should be chosen, in order to favour model interpretation and to avoid overfitting. In our approach to the hierarchical PLSR, both the global and the local regression models are kept for comparison. Hence, for each case of metamodelling one can evaluate the gain of using local modelling compared to the global model. The combination of local and global modelling is also useful for revealing regional differences in model sensitivity to the various parameters, e.g. by exploring the loading plots or regression coefficients for the local models. The local models also provide the opportunity to zoom into regions of the parameter space and identify the operative domains of the parameter space in an efficient manner.

The gain in prediction accuracy from using HC-PLSR was larger for metamodelling of the circadian clock data set than for the mouse ventricular myocyte data, implying that the circadian clock model exhibits behaviour which is harder to capture with linear polynomial models. This indicates that the circadian clock model contains more non-monotonicity or other complex nonlinear relationships between parameters and behaviour, but the systemic explanations are not easy to pinpoint as both dynamic models contain complex wiring. Even though polynomial PLSR and OLS gave reasonable predictions for many of the state variables in both the circadian clock and the mouse ventricular myocyte data set (see Figure 8 and Figure 11), the HC-PLSR results were superior to both PLSR and OLS in terms of metamodelling robustness. OLS gave slightly better results than global PLSR for both these data sets, since the rank was reduced in PLSR compared to OLS. Projecting the data into estimated latent variables and thereby decreasing the rank of the data has great advantages with respect to interpretation. Hence, there is a trade-off between interpretability and prediction accuracy. One may argue that the present criterion for choosing the number of components to include in the PLSR model (based on cross-validation) is a bit conservative in this case, since the regression modelling is based on noise-free data from deterministic models. Using HC-PLSR, we achieve a better prediction accuracy than both global polynomial PLSR and OLS, while keeping the advantages of reduced rank and increased overview and interpretability compared to OLS by using PLSR in the local modelling.

Our HC-PLSR using FCM clustering of the observations may be considered as a more or less automatic, "top-down" approach. In contrast, most existing hierarchical approaches are "bottom-up", that is, they start by estimating latent variables (PLSR scores) for individual, a priori known data blocks, and then combine these block scores into a second level linear PLSR model [16-19]. The use of fuzzy clustering to separate the observations into groups makes the HC-PLSR approach presented here applicable also in cases where prior knowledge about different blocks of data is not available. However, when such information is available, it should be used instead of clustering to separate the observations into groups. The number of clusters to use is here specified in advance. More computationally demanding alternatives exist, however, that allow automatic identification of the optimal number of clusters [50,51]. An alternative would be to include an automatic optimisation of the number of clusters (e.g. based on automatic exploration of several alternatives). This would allow for a semi-automatic metamodelling methodology which would be feasible for implementation e.g. as part of the CellML repository [7-9].

In contrast to crisp clustering methods, such as *K*-means clustering, which allocate each observation to a unique cluster, fuzzy clustering returns membership values for the different clusters for each observation [45]. This provides the opportunity to compute the regression coefficients for each observation as a weighted sum of the regression coefficients for the different clusters, where the membership values are used as weights. Predictions of response variables for the test set observations were here done both by a) selecting the local model for the most probable cluster and by b) computing the regression coefficients as a weighted sum of the local models. The first approach gave the best prediction accuracy in our case. The reason is probably that all data sets used in this paper were generated from experimental designs, and not by random sampling in the parameter space. For data sets with very distinct separations of the clusters, the first approach performs best, while for more continuous data, a weighted sum of the local models will probably be a better choice. Moreover, using a weighted sum will help avoiding discontinuities in regions where local models meet in more continuous data sets. We found that using only the first three PLS components in the FCM clustering gave the most distinct separation of the clusters for the three data sets tested here. This ensures that the most relevant information about the covariance between X and Y is being used for clustering when using the X-scores as basis. However, using only three PLS components may not be appropriate in general. For some data sets, one may want to use a larger number of PLS components. In order to use the Y-scores as a basis for clustering and classification, the separation of the observations needs to be quite distinct, since the Y-scores are based on predicted Y-values and therefore contain some prediction error that may disrupt the classification when the clusters are not distinctly separated.

OLS is less computationally demanding than both HC-PLSR and PLSR, and is a suitable metamodelling method in cases where the response surface is monotone, moderately nonlinear and the regressor variables are linearly independent. However, for the metamodelling approach to be feasible for automation, we need a robust regression method that can be automatically adjusted according to the properties of the response surface of the dynamic model to be emulated. HC-PLSR has advantages over other regression methods such as global PLSR and OLS in cases where the response surface is complex, highly nonlinear and non-monotone (in such cases, the model errors described by the RMSEP values from predictions with a global regression method will be much higher than the level of noise in the data), and the number of clusters used can be adjusted to suit the complexity of the response surface allowing for a robust automation of the metamodelling procedure.

## Conclusions

Our results show that it is possible to emulate dynamic models in systems biology to a high precision using multivariate regression, and that local modelling can improve the results substantially when the parameter to phenotype map is highly nonlinear. HC-PLSR was superior to both global polynomial PLSR and global polynomial OLS regression in all three model settings reported here. Since these model settings represent large classes of dynamic models and because the HC-PLSR method can be adjusted to suite the complexity of the dynamic model behaviour in a very flexible way inviting automation, it qualifies as a promising candidate approach for metamodelling within systems biology.

## Authors' contributions

KT contributed to conception, wrote the MATLAB^® ^code for the HC-PLSR pipeline, performed the data analysis and wrote the paper. UGI participated in designing and debugging of the HC-PLSR code, and participated in writing the paper. ABG performed the computational experiments with the gene regulatory network models and the mammalian circadian clock model, while JOV performed the computational experiments with the mouse ventricular myocyte model. SWO contributed with ideas for test cases and participated in writing the paper. HM contributed to conception and writing of the paper. All authors read and approved the final manuscript.

## Supplementary Material

Additional file 1 **'Additional file.pdf' contains Appendix 1, which provides background theory on the multivariate analysis methodology used, and Appendix 2-4 with supplementary figures and tables for each of the three test cases**. Click here for file
