# Supplementary material for: Hierarchical Cluster-based Partial Least Squares Regression (HC-PLSR) is an efficient tool for metamodelling of nonlinear dynamic models
Source: BMC Syst Biol. 2011 Jun 1;5:90. doi: 10.1186/1752-0509-5-90 (PMC3127793; doi:10.1186/1752-0509-5-90)
Supplement: Additional file 1 — 'Additional file.pdf' contains Appendix 1, which provides background theory on the multivariate analysis methodology used, and Appendix 2-4 with supplementary figures and tables for each of the three test cases. [file 1752-0509-5-90-S1.PDF]

Appendix 1. Multivariate analysis methodology

The most commonly used regression method, ordinary least squares (OLS) regression, is based on finding regression coefficients (b) that minimise the sum of squares of the residuals (f), given by equation A1,

$$y = b_0 + \sum_i x_i \cdot b_i + f = b_0 + Xb + f \qquad \text{(A1)}$$

where b_0 denotes the intercept.

Methods based on estimated latent variables, such as Principal Component Analysis (PCA) [21, 22] and Partial Least Squares Regression (PLSR) [23-25], are based on finding the directions in the X- and Y- variable space that explain the largest amount of the variation. While PCA finds the directions, represented by the score and loading vectors t and p, that maximise the variation in a matrix (X), PLSR maximises the covariance between the regressor matrix X and the response variable matrix Y. The PCA decomposition of the X-data into scores ($T_{x,PCA}$) and loadings (P_{PCA}) is given in equation A2, where A is the number of principal components used and $E_{A,PCA}$ is the residuals when using A components.

$$X = \bar{x} + \sum_{a=1}^{A} t_{xa} p_a' + E_A = \bar{x} + T_{x,PCA} P_{PCA}' + E_{A,PCA} \qquad \text{(A2)}$$

PLSR is a regression analogue to PCA, where both X and Y are decomposed simultaneously, maximising the covariance between X and Y. The PLSR version (SIMPLS) used in the MATLAB® [38] function "plsregress.m" (from the Statistics Toolbox™ v7.2) can be described as follows: The decomposition of X is given by equation A3a, while the decomposition of Y is given by equation A3b.

$$X = \bar{x} + T_x P' + E_A \qquad \text{(A3a)}$$

$$Y = \bar{y} + T_x Q' + F_A \qquad \text{(A3b)}$$

Q represents the Y-loadings and F_A is the Y-residuals when using A PLS components (PCs). Note that the score matrix T_x is the same in the X- and Y-decomposition. T_x is calculated by equation A4.

$$T_x = (X - \bar{x}) \cdot V = X_0 V \qquad \text{(A4)}$$

The PLSR loading weights, V, maximise the covariance between X and Y. The X- and Y-loadings (P and Q) are derived from equation A5,

$$P = X_0' T_x \qquad \text{(A5a)}$$

$$Q = Y_0' T_x \qquad \text{(A5b)}$$

where X_0 and Y_0 are the mean-centred X- and Y-matrices. The Y-scores (T_y) are initially computed by equation A6,

$$T_y = Y_0 Q = Y_0 Y_0' T_x \qquad \text{(A6)}$$

but each column of Ty is then orthogonalised with respect to the preceding columns of T_x, so that $T_x'T_y$ is lower triangular. The PLSR coefficients B_A at A PCs are given by equation A7.

$$B_A = V_A Q_A' \tag{A7}$$

V_A and Q_A denote the V- and Q- matrices truncated at A PCs. The response variables can then be predicted by equation A8, which is the PLSR analogue to equation A1.

$$Y = b_0 + XB_A + F_A \tag{A8}$$

In HC-PLSR, fuzzy *C*-means (FCM) clustering [44, 45] is used to separate the observations into clusters, for which local PLSR models are calibrated. In fuzzy cluster analysis a membership u_{ij} is defined for each object *i* and cluster *j*. The membership values are between 0 and 1, and must sum up to one for each object *i*. In FCM the membership values are found by minimising

$$J = \sum_{j=1}^{C}\sum_{i=1}^{N} u_{ij}^{m} d_{ij}^{2},\ m \geq 1 \text{ subject to } \sum_{i=1}^{N} u_{ij} = 1 \tag{A9}$$

Here d_{ij} is the Euclidean distance between object *i* and cluster *j* (i=1,2,…,N, j=1,2,…,C), *m* is a fuzzifier parameter that usually is set to be equal to 2.0. With m=1, FCM is the same as *K*-means clustering. *J* is minimised for a given $U=\{u_{ij}\}$ by setting the cluster centres v_j equal to the fuzzy means (see equation A10). Next, the membership values that minimise *J* for given distances $D=\{d_{ij}\}$ are calculated using equation A11. Then the *v*'s and the *d*'s are updated. This continues until convergence. The procedure is initialised randomly.

$$v_j = \frac{\sum_{i=1}^{M} u_{ij}^{m} x_i}{\sum_{i=1}^{M} u_{ij}^{m}} \tag{A10}$$

$$u_{ij} = \left(\sum_{k=1}^{C} \left(\frac{d_{ij}^{2}}{d_{ik}^{2}} \right)^{\frac{1}{m-1}} \right)^{-1} \tag{A11}$$

The basic FCM algorithm seeks spherical clusters. To find clusters with other shapes, modifications of the FCM algorithm must be applied, see for instance [50, 51].

Appendix 2. Gene regulatory networks (supplementary figure and table)

Figure A2.1 shows the data generated for the gene regulatory network motifs – 125 time series for each of the three state variables (X1-X3), integrated from 125 different initial conditions and recorded over 300 time steps for each motif.

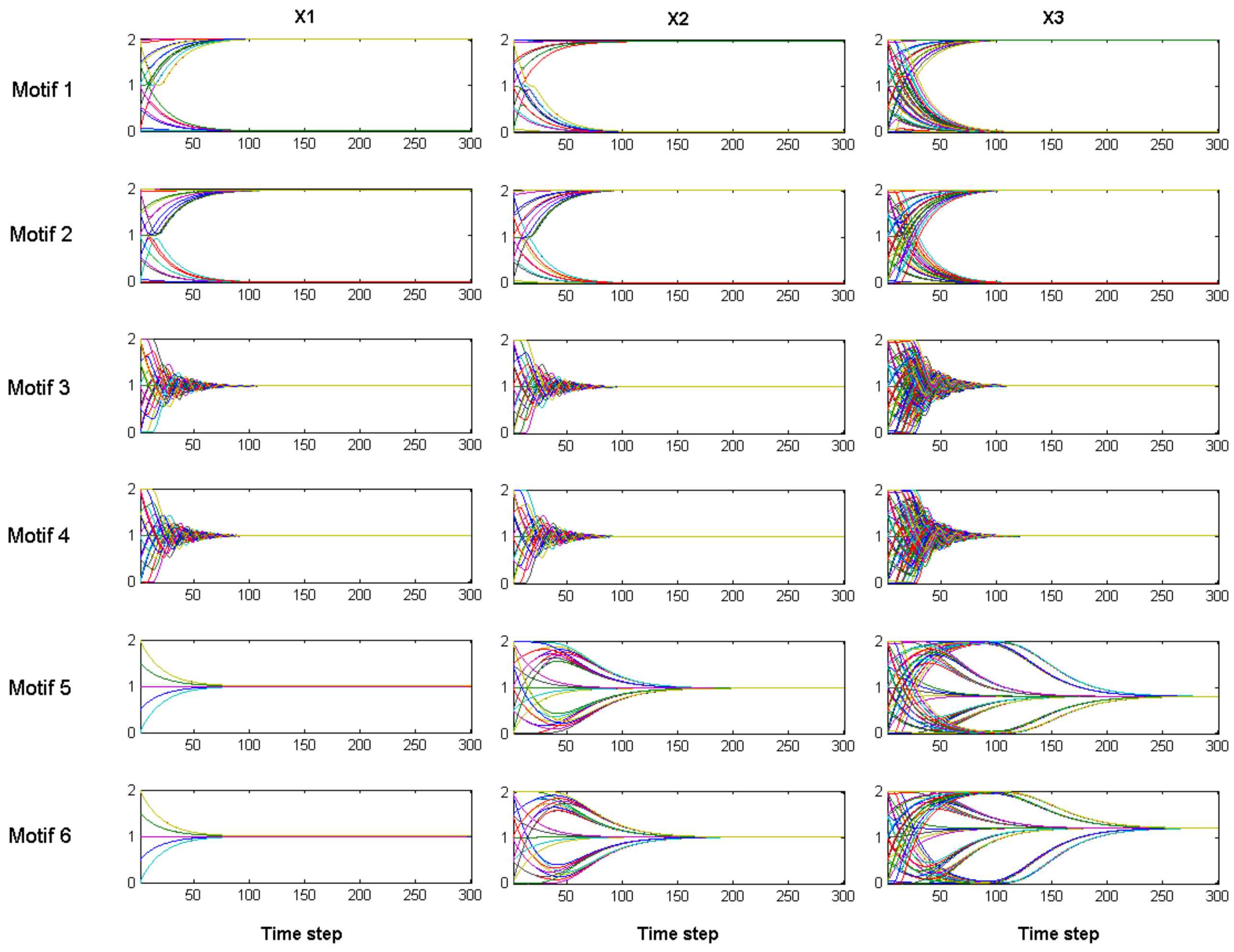

Figure A2.1. State variable time series for the six different gene regulatory network motifs. The time series correspond to 125 initial conditions for each of the three state variables (X1-X3). The parameter values of maximal production rates, decay rates and regulation thresholds were constant, and chosen to ensure that the steady state levels for all three variables were ranging from 0 (when the production rate is zero) to 2 for a maximal production rate.

The range within which the starting conditions for the three state variables X1, X2 and X3 varied in each cluster used in the HC-PLSR modelling of gene regulatory network motif 1 and 6 (corresponding to Figure 4 in the main text) are shown in Table A2.1.

Table A2.1. Clustering results used in HC-PLSR for gene regulatory network motif 1 and 6. The range for the starting conditions (X1_0, X2_0, X3_0) for the three state variables in the different clusters are shown.

Cluster	Range for X1_0	Range for X2_0	Range for X3_0
1, motif 1	0-2	0-2	0-2
2, motif 1	0-1.5	0.5-2	0-2
1, motif 6	0-0.5	0-2	0-2
2, motif 6	1	0-2	0-2
3, motif 6	1.5-2	0-2	0-2

Appendix 3. Mammalian circadian clock (supplementary figures)

The state variable time series from the simulations of the mammalian circadian clock are shown in Figure A3.1.

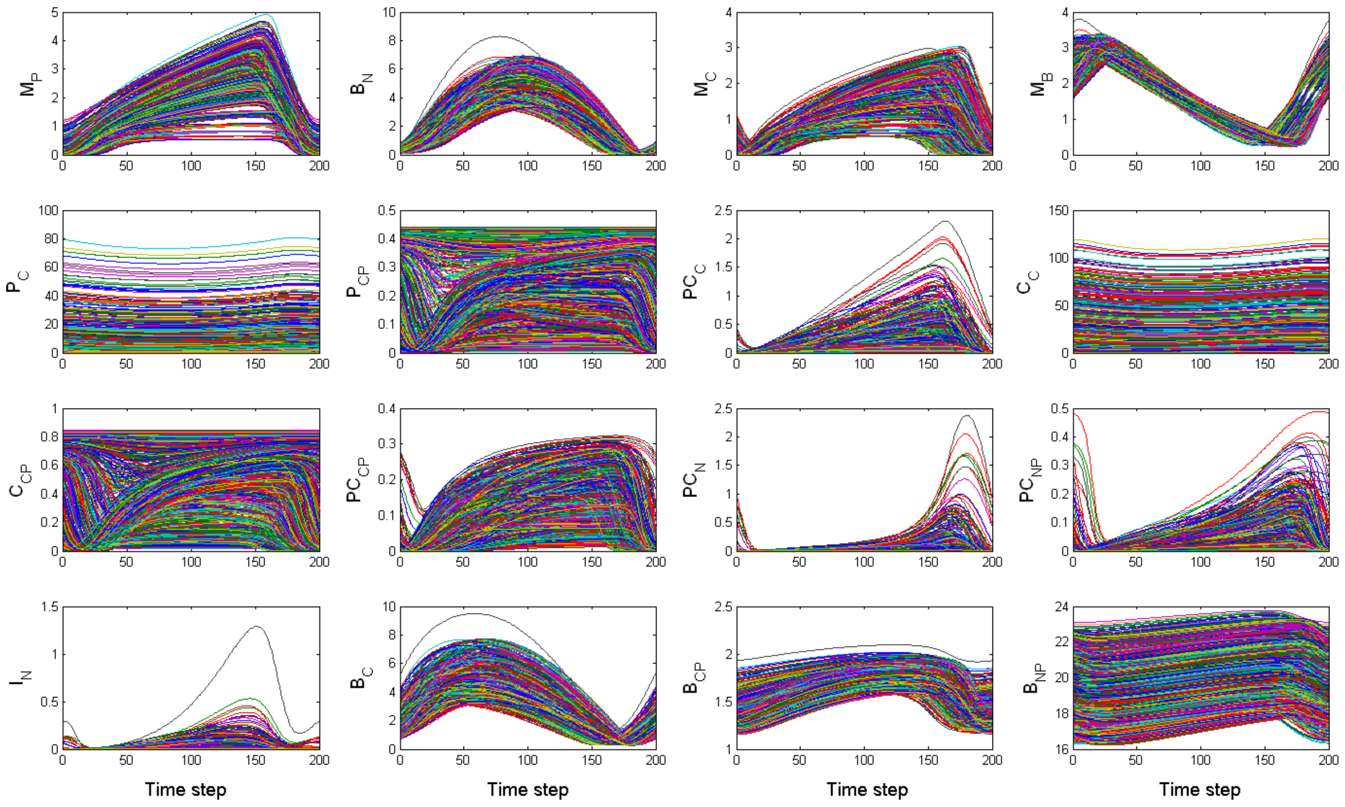

Figure A3.1. State variable time series for the mammalian circadian clock. Each curve represents one set of parameter values simulated over 200 time steps.

The optimal number of clusters to use in the HC-PLSR was chosen based on predictions using the observations in the calibration set as if they were "new observations", that is, the same procedure as for the test set was used. The results are given in Figure A3.2.

A) B)

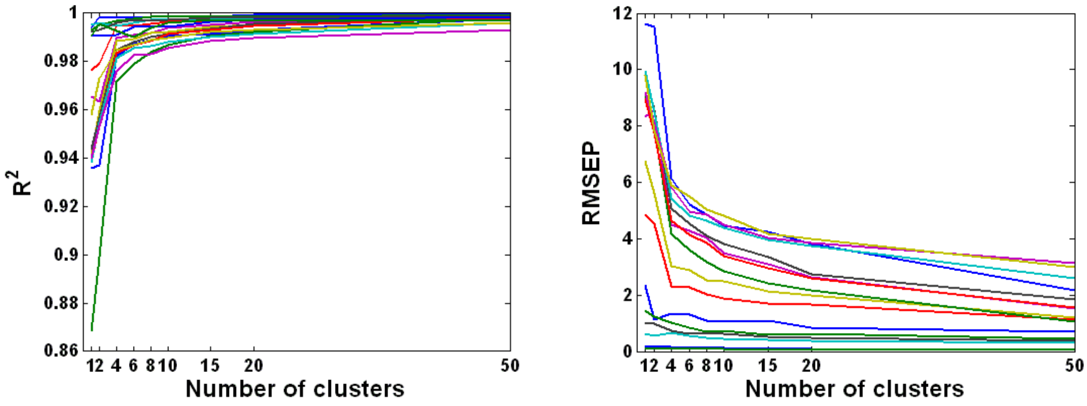

Figure A3.2. Selection of the optimal number of clusters in HC-PLSR for the mammalian circadian clock. A) R^2 and B) RMSEP values from calibration set predictions of the circadian clock time series using from 1 to 100 clusters in HC-PLSR. Each curve represents one particular state variable. One cluster is equivalent to ordinary PLSR. The X-axes were truncated in order to increase the overview.

The mean prediction residuals from the test set predictions with HC-PLSR are shown in Figure A3.3 as a function of the distance from the nearest cluster.

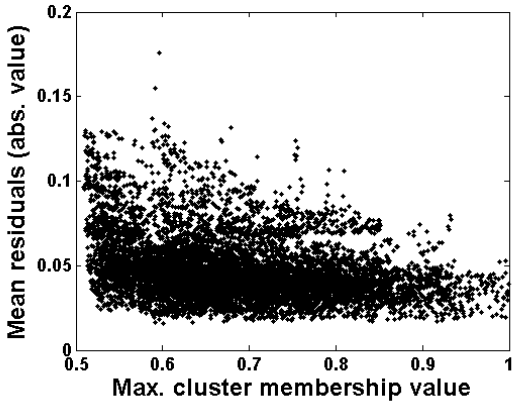

Figure A3.3. Prediction Y-residuals vs. distance to nearest cluster in HC-PLSR for the state variable M_P. Mean residuals from HC-PLSR (absolute value) taken over all calculated time steps for the circadian clock state variable M_P plotted against the maximum obtained cluster membership value for each test set observation. The cluster membership values are based on the Euclidian distance to the clusters.

Prediction accuracy for the mammalian circadian clock obtained using HC-PLSR with local modelling of the entire Y-matrix is compared to that obtained using local modelling of only the Y-residuals from the global modelling in Figure A3.4.

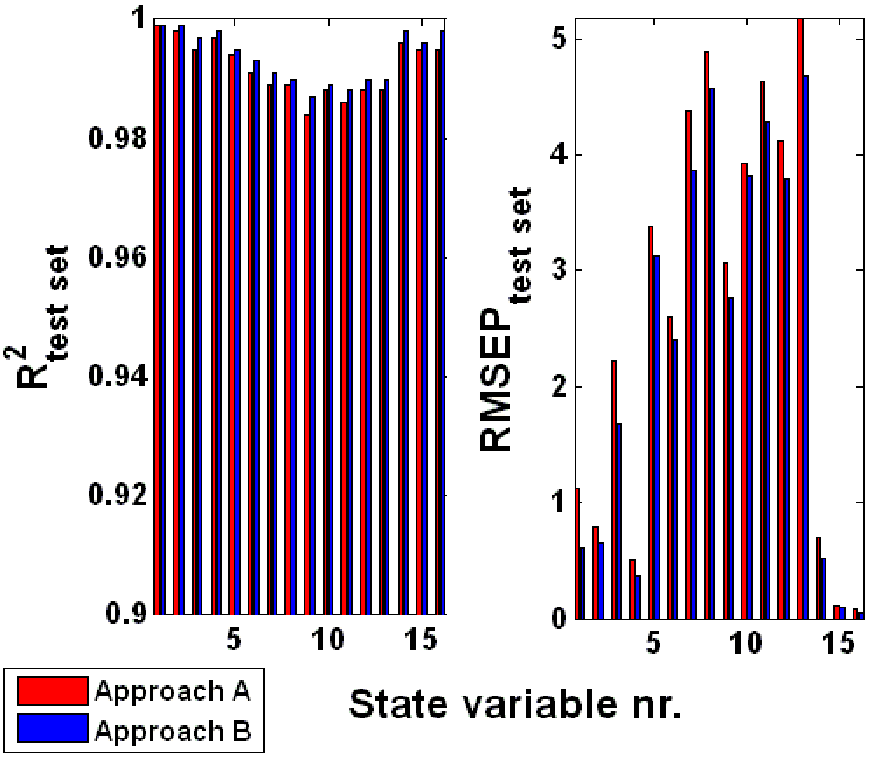

Figure A3.4. Comparison of two alternative HC-PLSR variants. The prediction accuracy obtained for the mammalian circadian clock data using HC-PLSR with local modelling of the Y-matrix (approach A) is compared to that obtained using HC-PLSR with local modelling of only the Y-residuals from the global modelling (approach B). The same regressor matrix was used in both cases.

Appendix 4. Mouse ventricular myocyte (supplementary figures)

A subset of the state variable time series for the mouse ventricular myocyte is shown in Figure A4.1.

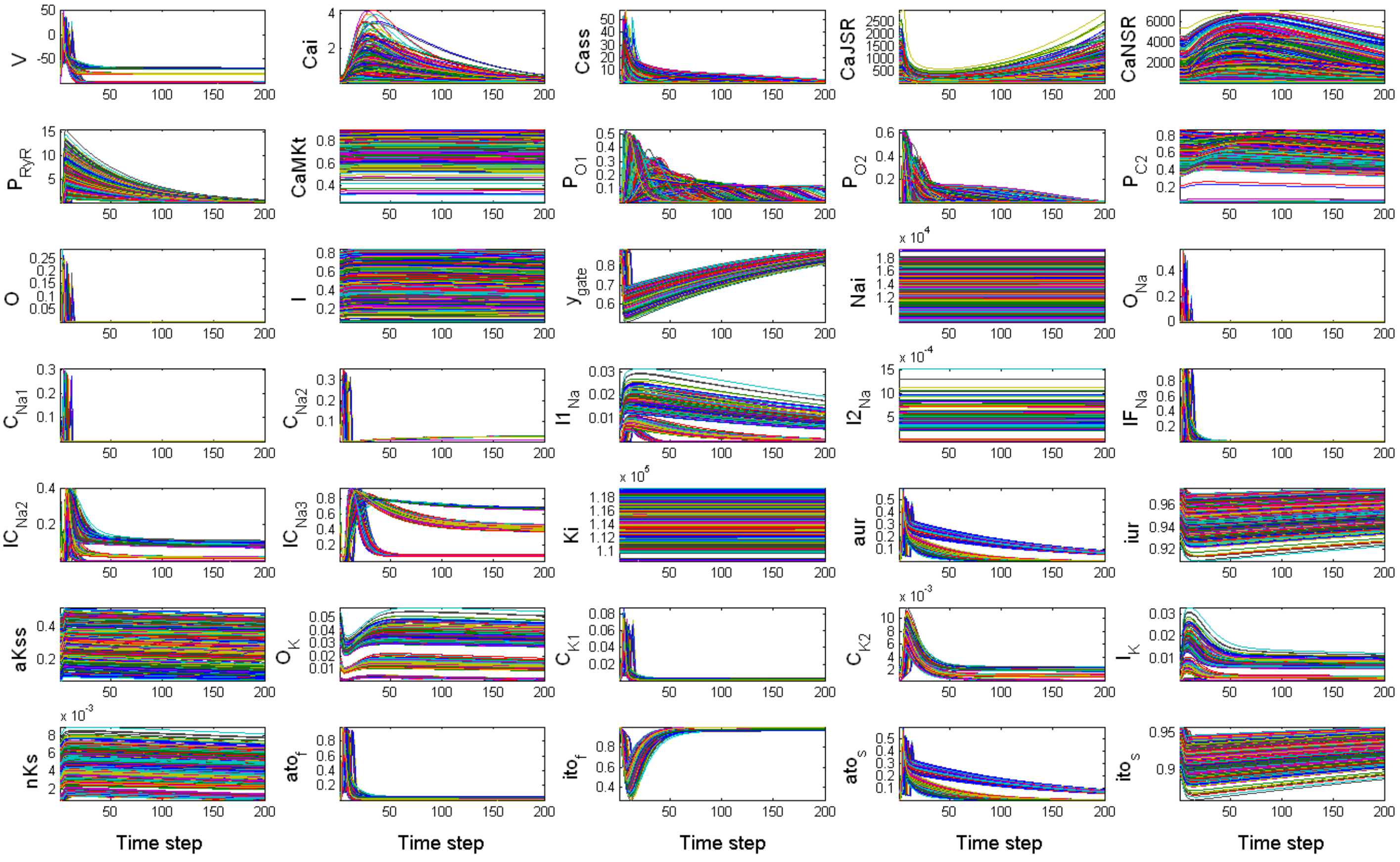

Figure A4.1. State variable time series for the mouse ventricular myocyte. The state variable time series for the mouse ventricular myocyte for 1000 randomly chosen simulations that converged. Data for the 200 interpolated time steps, within the 333.33 ms stimulus period data set is shown.

The optimal number of clusters to use in the HC-PLSR was chosen based on predictions using the observations in the calibration set as if they were "new observations". The results are given in Figure A4.2.

A) B)

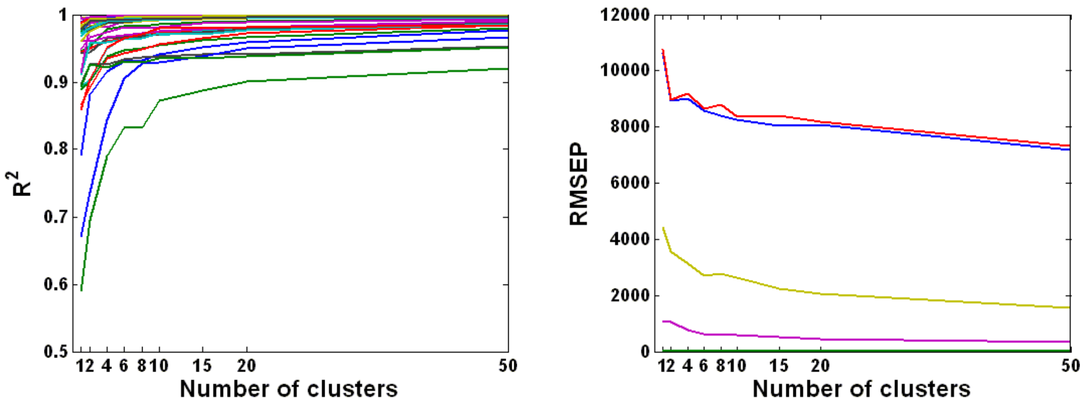

Figure A4.2. Selection of the optimal number of clusters in HC-PLSR for the mouse ventricular myocyte. A) R^2 and B) RMSEP values from calibration set predictions of the mouse ventricular myocyte time series (333.33 ms stimulus period data set) using from 1 to 100 clusters in HC-PLSR. Each line represents one particular state variable. One cluster is equivalent to ordinary PLSR. The X-axes were truncated since RMSEP increases enormously when the number of clusters is larger than 50.

The mean prediction residuals from the test set predictions with HC-PLSR are shown in Figure A4.3 as a function of the distance from the nearest cluster.

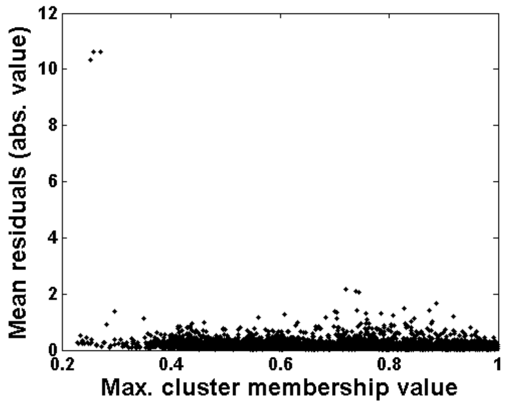

Figure A4.3. Prediction Y-residuals vs. distance to nearest cluster in HC-PLSR for the state variable V. Mean residuals from HC-PLSR (absolute value) taken over all calculated time steps for the mouse ventricular myocyte state variable V (the action potential) against the maximum obtained cluster membership value for each test set observation (333.33 ms stimulus period data set). The cluster membership values are based on the Euclidian distance to the clusters.
